# Acetabular Home Run Screw Guidance for Transiliac Fixation in Cup Revision Arthroplasty

**DOI:** 10.3390/jcm14030922

**Published:** 2025-01-30

**Authors:** Martin Wessling, Carsten Gebert, Mohamed Marei, Marcel Dudda, Arne Streitbuerger, Mirko Aach, Lee Jeys, Sven Frieler, Daniela Koller, Yannik Hanusrichter

**Affiliations:** 1 Department of Tumor Orthopedics and Revision Arthroplasty, Orthopedic Hospital Volmarstein, 58300 Wetter, Germany; wesslingm@esv.de (M.W.); gebertc@esv.de (C.G.) frielers@esv.de (S.F.); hanusrichtery@esv.de (Y.H.); 2 Center for Musculoskeletal Surgery, University Hospital of Essen, 45147 Essen, Germany; marcel.dudda@uk-essen.de (M.D.); arne.streitbuerger@uk-essen.de (A.S.); 3 Department of Orthopedics and Tumor Orthopedics, Muenster University Hospital, 48149 Muenster, Germany; 4 Department of Trauma Surgery, University Hospital Essen, 45147 Essen, Germany; 5 Department of Orthopedic Oncology, University Hospital Essen, 45147 Essen, Germany; 6 Department of Trauma and Orthopedic Surgery, BG University Hospital Bergmannsheil, Ruhr-University Bochum (RUB), 44801 Bochum, Germany; mirko.aach@bergmannsheil.de; 7 Spinal Cord Injury Unit, Department of General and Trauma Surgery, BG University Hospital Bergmannsheil, Ruhr-University Bochum, 44879 Bochum, Germany; 8 Oncology Department, The Royal Orthopedic Hospital, Birmingham B31 2AP, UK; lee.jeys@nhs.net; 9 Faculty of Health Sciences, Aston University, Birmingham B4 7ET, UK; 10 Institute of Medical Information Processing, Biometry, and Epidemiology, Faculty of Medicine, LMU Munich, 80539 Munich, Germany; daniela.koller@med.uni-muenchen.de

**Keywords:** hip arthroplasty, revision arthroplasty, home run screw, transiliac fixation, screw angle

## Abstract

**Background/Objectives:** The growing incidence of acetabular revisions has highlighted the importance of achieving reliable fixation to the remaining bone. Proximal transiliac fixation (TIF) of pelvic implants is becoming an increasingly common approach for managing extensive bone defects. This study seeks to provide guidance on TIF implantation by analyzing the optimal screw placement in partial pelvic replacements for acetabular defects. **Methods**: Between 2014 and 2024, a cohort of 96 consecutive patients (65 females and 31 males) who underwent customized partial pelvic replacement (PPR) with transiliac fixation (TIF) were examined. The angle and entry point of the ideal TIF were determined using preoperative three-dimensional planning and compared with potential influencing factors. **Results**: All PPRs were successfully implanted, with an average TIF length of 77 mm. The mean anteroposterior angle for TIF was 18° medially and 27° dorsally. **Conclusions**: Analysis of the entry point showed concentration around the second radius and between the eleven o’clock and one o’clock positions. The AP angle is notably affected by gender and height. Considering the precision of human judgment, a recommendation for TIF placement would be 20° medial and 30° dorsal deviation, with the entry point around the twelve o’clock position and the second ring from the center of the cup.

## 1. Introduction

Hip arthroplasty is widely regarded as one of the most successful procedures in orthopedics. However, with the increasing availability of arthroplasty procedures and longer life expectancy, there has been a parallel rise in the need for revisions and multiple revision surgeries of the hip joint [1]. Complex acetabular defects have driven the development of various treatment options for metal-based reconstruction of the hip joint [2]. As part of an international consensus symposium on acetabular bone loss, Sculco et al. published a comprehensive review of the current recommendations for the diagnosis, classification, and treatment of acetabular defects in 2022 [3]. The outcomes of such complex reconstructions, using highly porous revision implants, with or without augmentations, cages, pedestal cups, or customized partial pelvic replacements (PPRs), are highly satisfactory and have gained widespread acceptance. Recent meta-analyses by Malahias et al. suggest that cementless restoration using modular macroporous implants is superior to isolated mechanical cage reconstruction [4]. However, in all cementless metal reconstructions, additional screw fixation is necessary to ensure secondary osseointegration, especially in cases where the primary press fit is compromised [5]. Mechanical analyses suggest that an adequate screw fixation length in the primary weight-bearing orientation plays a crucial role in improving the stability of the reconstruction [6].

In the analyses by Wasielewski et al. regarding the safe zone of this screw fixation, the acetabulum is divided into quadrants, and placement in the posterior superior quadrant is recommended as a safe zone. In contrast to this, posterior inferior orientation is defined as a caution zone [7,8]. However, as superior defects are more commonly encountered, screw placement, especially of a sufficient length, becomes increasingly difficult, and is often only possible in the caution zone, with the risk of damaging the sciatic or superior gluteal nerve. This is aggravated by the fact that the entry point for a corresponding screw must be selected in such a way that the screw can still be placed with the cup in a favorable position. Additionally, Smitham, PJ. et al.’s study identified the potential risk of injury to the gluteal neurovascular bundle associated with the use of cage implants for iliac fixation [9]. To facilitate secure cup fixation, there has been growing research into the additional use of inferior fixation via the os pubis and ischium [10].

The positive results achieved with iliacal fixation using pedestal cups have led to the adaptation of long transiliac fixation (TIF) for custom-made implants and newer revision systems [11]. The aim of a TIF is to achieve a long-lasting secure anchorage in the iliac bone in the main load-bearing direction. It has been shown that in the case of extensive defects and the use of customized pelvic partial replacements with proximal fixation principles, a transiliac fixation of generally more than 60 mm in length and 8 mm in diameter (‘home run screw’) can be used without inferior fixation [12].

However, achieving the same length of transiliac fixation with off-the-shelf implants presents a greater challenge in freehand preparation. Using 3D planning in comparison to 2D planning, a study by Brandt et al. was only able to achieve an average screw length of 50.5 mm in the ilium [13]. Unlike customized pelvic replacements, off-the-shelf implants do not permit the use of 3D-planned screw orientation or patient-specific drill guides [12]. As a result, the entry point is often predetermined by the implant design, which may not be optimally positioned. While anatomical studies in the literature recommend using the sciatic notch as a landmark for placing a home run screw [14], there is a lack of clinical guidance on optimal angles and entry points for this procedure.

The most complex planning for one or two home run screws is carried out during the creation of custom-made PPRs, and thus provides a valuable data basis for anatomical analysis. Due to the satisfying experiences with PPR, the planned angles and entry points of TIF were analyzed in successfully performed procedures to facilitate their application with off-the-shelf implants.

The objective of this study is to determine the optimal angle for transiliac fixation in cup revision surgery, and to identify the ideal entry point for the fixation screw. In addition, the study aims to investigate whether there are any factors that influence this angle and, if so, which ones. By analyzing these results, the study aims to generate recommendations as well as reproducible anatomical analysis yielding surgical guidelines, while also taking the published results into account.

## 2. Materials and Methods

**Figure 1:**
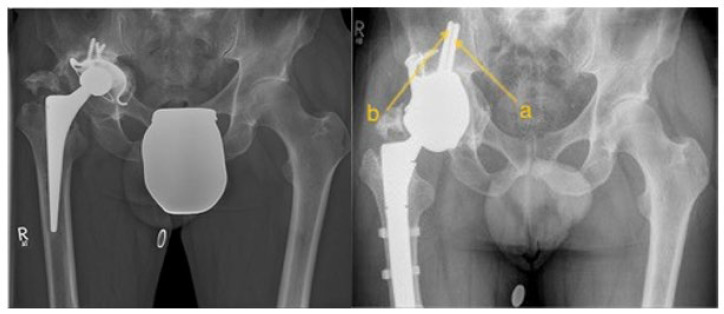
Illustration of the preoperative condition on the left side and the postoperative outcome after PPR with transiliac fixation on the right side. (**a**) TIFp, (**b**) TIFs.

All consecutively planned individual pelvic replacements between 2014 and 2024 in a reference center for revision surgery were retrospectively analyzed. In all successfully implanted PPRs, the digital planning was analyzed to determine the optimal TIF. All planning was carried out in close collaboration with the prosthesis manufacturer’s engineers from Implantcast GmbH, Buxtehude Germany and approved by MW, and from 2020, always by two of the surgeons involved. The successful implantation was performed by surgeons MW, CG, and YH (see Figure 1).

**Figure 2:**
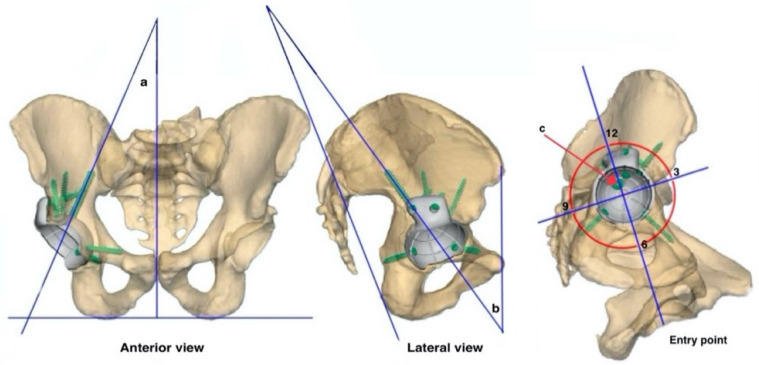
Exemplary illustration of the measured angles and entry point. (**a**) The AP angle, which reflects the lateral deviation from the midline in anterior view of the pelvis. (**b**) The lateral angle of the transiliac fixation in lateral view of the pelvis. (**c**) The entry point of the screw in the surgeon’s view, in this case 112.

The three-dimensional dataset created during planning using Geomagic Freeform Plus (Version 2019.2.50, 3D-Systems, Moerfelden-Walldorf, Germany) and Simpleware ScanIP Medical (Version R-2020.09, Synopsys, Sunnyvale, CA, USA) was standardized in an anterior, lateral, and isometric (surgeon’s view) view. The strictly anterior plane was defined [15] and a line was drawn along the ischial tuberosities for the measurements. The inclination and anteversion of the acetabulum and the angle of the transiliac fixation to the orthogonal and acetabular inclination were then determined for this plane (see Figure 1). The lateral view was aligned as a strict superimposition of the pelvis in lateral view and the anterior pelvic plane between the anterior superior iliac spine and the symphysis pubis was drawn. The transiliac fixation was determined for this plane (see Figure 1). The angle between the anterior pelvic plane and posterior superior iliac spine and ischial bone (lateral pelvic angle) is used as a predictor for the relative size of the pelvis (see Figure 1). The isometric view was chosen as the surgeon’s view of the acetabulum, with the best possible visualization of the transiliac fixation entry point. A clock was placed on it for orientation. The alignment was based on the anatomical landmarks, with 6 o’clock at the center of the transverse ligament [16] (see Figure 1). For a consistent position between the left and right pelvis, the clock was mirrored at the midline, so that 12 o’clock always pointed proximally and 3 o’clock always pointed ventrally. The planning allowed a radial division based on the given acetabular planes, with five different height localizations (see Figure 2). Based on this consideration, the entry point for the transiliac fixation was identified based on its position relative to time (using a clock-like representation) and its radial distance from the acetabular base. These two parameters were combined to define an entry field (see Figure 1). When aligning the pelvis in the standard planes, there were overlaps, so the AP angle could not be measured in 0.7% (*n* = 1), nor the lateral angle in 2.6% (*n* = 4). The entry point for the 151 TIFs could not be accurately defined in 9.3% (*n* = 14) due to the overlap.

In case of the two planned transiliac fixations, the parameters were clearly specified. The sciatic notch angle, being the closer reference point, was the primary determining factor for TIFp, and the second screw as TIFs. In addition, the cup size and the length of the transiliac fixation were recorded.

Demographic data were also collected for all patients. Due to anatomical differences between the female and male pelvis, special consideration was given to gender-specific groups. In addition, the influencing factors of age, defect size, pelvic discontinuity, height, weight, BMI, lateral pelvic angle, and length of transiliac fixation on the angle of transiliac fixation were analyzed.

All patients agreed to the described method, giving informed consent and permission to submit their data for analysis. Ethical approval was obtained from the local ethics committee (reference number 21-10438-KOBO) prior to the investigation.

### Statistical Analyses

The Shapiro–Wilk test was performed to determine non-normal/normal distribution. If not mentioned otherwise, results are stated as mean + standard deviation (range). Descriptive statistics are shown for all screws, and also separately for patients receiving only one or two screws.

To identify potential factors influencing the angles, an exploratory multivariate linear regression model was conducted for each outcome angle (AP and lateral). Given the limited evidence in the existing literature, we initially applied a full model that included all potential predictors, followed by a backward stepwise approach, to achieve the best model fit.

Multicollinearity was assessed using the Variance Inflation Factor (VIF) for multivariate regression. A VIF value below 5 was considered acceptable and indicative of no problematic collinearity.

All data analysis was performed using R Studio Version 2024.04.2.

## 3. Results

A total of 97 individualized pelvic replacements were evaluated in 96 patients, comprising 65 females and 31 males. The analysis included the following variables: gender, age, acetabular defect, pelvic discontinuity, screw length, posterior pelvic angle, entry point, and the number of screws.

The mean age at the time of surgery was 70.5 years. All planned pelvic replacements were successfully implanted. The key parameters, including defect size, pelvic discontinuity, height, weight, BMI, cup alignment, lateral pelvic angle and transiliac fixation lengths, are summarized in (Table 1).

In total, 151 transiliac fixations (TIFs) were analyzed. Isolated fixation in the primary position (TIFp) was performed in 43 cases of primary pelvic replacement (PPR), while additional secondary fixation (TIFs) was used in 54 cases. The average length was 79 mm for TIFp and 75 mm for TIFs. Overall, the transiliac fixation demonstrated a medial angle of 18° in the anterior view, and a lateral angle of 27° relative to the anterior pelvic plane in the lateral view (see Table 2).

**Figure 3:**
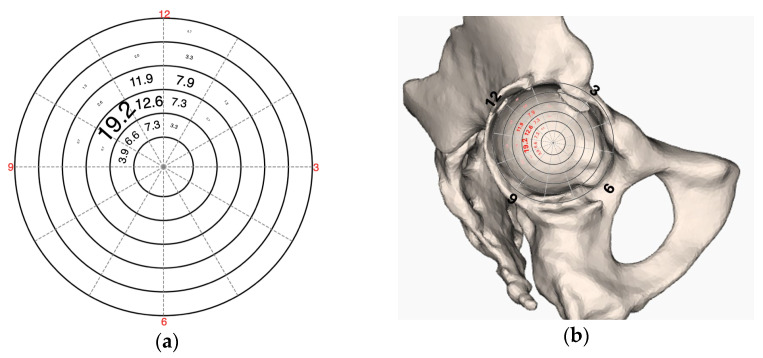
(**a**) A plot illustrating a clock face, with the statistically detected entry pointsall examined TIFs. (**b**) a 3D anatomical rendering of the pelvic bone, demonstrating the spatial distribution of the entry points.

Analysis of the point of entry revealed that the second radius exhibited the highest frequency at 40.4%, followed by the third radius at 25.2% and the first radius at 18.5%. When evaluating entry point based on the clock face orientation, 12 o’clock was the most frequent position, observed in 33.8% of cases, followed by 11 o’clock at 30.5% and 1 o’clock at 23.2%. Additional details and the distribution of values between primary (TIFp) and secondary (TIFs) fixation are presented in Table 2. To illustrate entry point, Figure 3 shows the sum of the entry fields in percentage for all TIFs in a network diagram and in relation to a pelvis.

**Figure 4:**
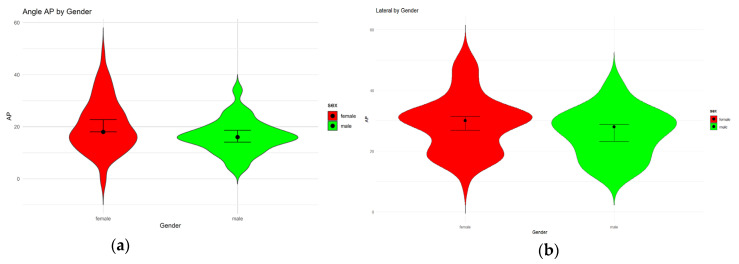
Boxplot plots illustrating the distribution of the AP angle (**a**) and lateral angle (**b**) by gender. The red plots represent female participants, while the green plots represent male participants.

In bivariate analysis, gender showed to have a significant effect on the AP angle (*p*(*t*-test): 0.0164) but not on the lateral pelvis angle, despite a strong trend (*p*(*t*-test): 0.0884). The distribution of both angles does however vary in both genders (see Figure 4).

To determine possible combinations of effects, a multivariate linear regression was performed for the AP and lateral pelvis outcome angles.

Because of a lack of evidence from the literature, all potential predictors were included in a full model, which was then adjusted to the best model fit with backward stepping. The results of the final models are shown in Table 3.

The lateral pelvic angle was the only variable with a prediction value for both outcome angles (however, it was only significant for the lateral angle). For the AP angle, the height of the patient, cup size, and Paprosky defect type also showed a correlation.

## 4. Discussion

In this study, the orientation and optimal entry point for long transiliac fixation (TIF) were demonstrated for the first time in a larger cohort. Clinically, these fixations play a crucial role in individualized pelvic reconstructions [17]. In an experimental study by Jaenisch, M. et al. on the primary stability of modular pelvic reconstruction implants, it was also shown that screw fixation achieved high primary stability, whereby three iliac screws of 25, 50, and 60 mm were used. However, the working group has identified a supplementary strap as an additional advantage for the stability of the shell [18]. The current literature shows an increasing use of modern macroporous reconstructions of the pelvis and a reduced use of cages in such reconstructions [4].

It is important to highlight that the favorable outcomes observed in PPR are likely influenced not only by the length of the TIF, but also by its thickness. Additionally, 58 of the transiliac fixations discussed here are performed using modular stems, which have a larger diameter of 9 mm compared to conventional screws and facilitate osteointegration. When using thinner screws, a greater deviation in the angles is possible without causing a screw misplacement. The studies by Wasielewski et al. provide a good overview of the placement and lengths of screws in the normal and dysplastic pelvis [7,8]. Essentially, these studies specified the zones and in some cases the entry points for screws of up to 35 mm in length. It is well documented in previous studies which risks exist for vessels and nerves in the case of screw misplacement. The TIF mentioned in the work is of particular interest for modern revision surgery, considering that actual angle and entry point analyses for these fixations have not been carried out [7,8].

However, the work of Kaplan et al. shows that a significant increase in stability can already be expected with screws of 50 mm in length [5]. It is therefore not unlikely that stability will also increase further with even longer screws, or that a simultaneous increase in the thickness of the screw will occur. Another effect may be the transmission of the acting forces along the screw, similar to the mechanical stability of the pelvic ring [19]. Other effects that influence screw fixation are eccentric placement or transcortical or cancellous placement of the screw [20,21].

The quantification of the entry point for transiliac fixation shown here enables a straight line to be drawn, despite the cup being in an anatomical position for the preparation and implantation of screws longer than 60 mm. These entry points correspond relatively well with the entry points given in Wasielewski’s work, but include significantly more central and posterior fields. The overview given here can therefore be seen as a new and more comprehensive guide to entry point, for surgeons to perform a TIF among extensive acetabular defects. However, it must be checked for each individual case whether the soft tissues allow for appropriate preparation and implantation. The transferability of the results shown here to freehand preparation of transiliac fixation of implants in the sense of a home run screw has been confirmed in initial publications with newer implants [22]. However, the use of the guidelines shown here based on anatomical landmarks is also recommended for other implant systems, particularly in combination with larger augments.

For the clinical transfer of the statements made here on the angles and analysis of the factors influencing the angle, the angle changes that can still be recognized by a surgeon must be seen. The analysis by Woerner et al. showed that even experienced surgeons can determine the angle of hip endoprosthesis with an accuracy of visual judgment of approx +/−5° [23]. The subsequent surgical feasibility of implantation from planning to realization is important for the interpretation of the clinical transferability of the angles determined here. By evaluating the accuracy of implant placement based on pre-surgical planning, an earlier study by the working group showed an average deviation of less than 5° for 45 of the patients examined here [24]. The deviation falls within the range of angles typically associated with the accuracy of the surgical eye, demonstrating that the planning conducted for the TIF can be executed with a high degree of reliability.

The analysis of the factors influencing the angles reveals that the AP angle is affected by gender, cup size, and body height.For the lateral angle, there is only a significant effect for the lateral pelvic angle. Due to the above-mentioned accuracy of the surgical eye and the assumed changes in the angles depending on the predictor, the clinical relevance for the surgeon is questionable. If necessary, larger collectives should be analyzed to determine whether other predictors are noteworthy, or whether the predictors selected here have a greater influence.

There are hardly any clinical data in the literature on the length of iliac screw fixations. Looking at the literature available here, a screw length of more than 60 mm has not usually been achieved, even under laboratory conditions [5,22,25]. In contrast, the average possible screw length for a home run screw in our cohort is 77 mm. Across all studies, the implant is typically stabilized against rotation using two additional screw fixations, which are often shorter than the “home run screw”, as stated in the study by Jaenisch et al. [18].

It can therefore be assumed that the recommendations made here for the angles of 18° to the medial and 27° to the posterior can be taken as robust recommendations for surgical practice. It is also noteworthy that in the present work, considering these angles and the entry points, two TIFs could be placed in over 50% of the cases. It can therefore be assumed that with the guidance provided, at least a long TIF is also possible freehand for most treatment options.

For the sake of simplicity, we therefore recommend the values with a deviation of TIF of 20° to the medial and 30° to the dorsal for surgical practice.

## 5. Conclusions

The TIF “home run screw” is one of the most important methods for the proximal securing of pelvic reconstruction in revision arthroplasty. As a guideline for placement, the entry point should be placed at approximately twelve o’clock, and on the second radius from the center. The angle of the home run screw should deviate approximately 20° medially and 30° dorsally. It does not appear necessary to adjust the angles for the risk factors examined.

## 6. Limitations

Several limitations must be taken into account for this study. First and foremost, very large and complex heterogeneous pelvic defects were retrospectively investigated, and the number of cases may not be sufficient to allow for general transferability. Furthermore, although the COR was selected and mirrored as closely as possible to the contralateral side, and an implant was designed accordingly, the representation of the inner side of the cup analog to a reamed cup is an assumption for these defects, and may differ depending on the individual preparation of the off-the-shelf implant.

## Figures and Tables

**Table 1:** Analysis of the main considerable factors.

Parameter	Value
Mean age, yrs (SD; range)	70.5 (11.6; 40 to 89)
Female, *n* (%)	65 (68.0)
Side left, *n* (%)	44 (45.4)
Mean BMI, kg/m^2^ (SD; range)	28.4 (6.6; 17 to 45)
Cup inclination (SD; range)	43.8 (3.7; 40 to 45)
Cup anteversion (SD; range)	18.7 (2.7; 15 to 20)
Paprosky defects, *n* (%)	
2a	1 (1.0)
2b	3 (3.1)
2c	3 (3.1)
3a	17 (17.5)
3b	73 (75.3)
Pelvic discontinuity, *n* (%)	39 (40.2)
Mean cup size (SD; range)	48 (4.1; 32 to 56)
Mean lateral pelvic angle (SD; range)	14.8 (2 to 31)
9 mm modular stem, *n* (%)	58 (38.4)
8 mm screw, *n* (%)	74 (49.0)
6.5 mm screw, *n* (%)	19 (12.6)

**Table 2:** Table of angles and entry points divided into all TIF, TIFp, and TIFs.

Parameter	All TIF (*n* = 151)	Only TIFp (*n* = 97)	Only TIFs (*n* = 54)
Mean AP angle (SD; range) (°)	18.3 (8.6; 0 to 48)	19.1 (9.0; 0 to 48)	16.9 (7.5; 1 to 44)
Mean lateral angle to pelvic plane (SD; range) (°)	27.3 (8.9; 9 to 52)	28.2 (9.0; 9 to 52)	25.7 (8.5; 13 to 51)
Mean screw length (SD; range) (mm)	77 (13.0; 45 to 100)	79 (13.1; 50 to 100)	75 (12.2; 45 to 100)
Entry point radius *n* (%)			
1	28 (18.5)	24 (24.7)	4 (7.4)
2	61 (40.4)	43 (44.3)	18 (33.3)
3	38 (25.2)	17 (17.5)	21 (38.9)
4	10 (6.6)	3 (3.1)	7 (13.0)
5	1 (0.7)	0 (0)	1 (1.9)
Entry point clock face *n* (%)			
1	35 (23.2)	14 (14.4)	21 (38.9)
2	3 (2.0)	0 (0)	3 (5.6)
10	4 (2.6)	2 (2.1)	2 (3.7)
11	46 (30.5)	42 (43.3)	4 (7.4)
12	51 (33.8)	30 (30.9)	21 (38.9)
Entry field *n* (%)			
101	5 (3.3)	5 (5.2)	0 (0)
110	2 (1.3)	0 (0)	2 (3.7)
111	10 (6.6)	11 (11.3)	0 (0)
112	11 (7.3)	8 (8.2)	2 (3.7)
201	11 (7.3)	4 (4.1)	7 (13.0)
202	1 (0.7)	0 (0)	1 (1.9)
210	1 (0.7)	1 (1.0)	0 (0)
211	29 (19.2)	26 (26.8)	3 (5.6)
212	19 (12.6)	12 (12.4)	7 (13.0)
301	12 (7.9)	5 (5.2)	7 (13.0)
302	2 (1.3)	0 (0)	2 (3.7)
310	1 (0.7)	1 (1.0)	0 (0)
311	4 (2.6)	3 (3.1)	1 (1.9)
312	18 (11.9)	8 (8.2)	10 (18.5)
401	5 (3.3)	0 (0)	5 (9.3)
411	2 (1.3)	2 (2.1)	0 (0)
412	3 (2.0)	1 (1.0)	2 (3.7)
501	1 (0.7)	0 (0)	1 (1.9)

**Table 3:** Analysis of the remaining predictors for the AP and lateral angle after backward stepping model fit.

AP Angle				
	estimate	std.error	statistic	*p*-value
(Intercept)	37	20.5	1.81	0.0745
Height	−20.9	9.16	−2.29	0.0249
Acetabular cup	0.398	0.201	1.98	0.0512
Paprosky defect type	−1.85	1.13	−1.64	0.105
Lateral pelvic angle	0.242	0.151	1.6	0.113
**Lateral angle**				
Term	estimate	std.error	statistic	*p*-value
(Intercept)	11.3	1.66	6.79	>0.0001
Lateral pelvic angle	1.11	0.103	10.8	>0.0001

## Data Availability

The datasets used and/or analyzed during the current study are available from the corresponding author upon reasonable request.
